# A Kinetic Model Considering Catalyst Deactivation for Methanol-to-Dimethyl Ether on a Biomass-Derived Zr/P-Carbon Catalyst

**DOI:** 10.3390/ma15020596

**Published:** 2022-01-13

**Authors:** Javier Torres-Liñán, Ramiro Ruiz-Rosas, Juana María Rosas, José Rodríguez-Mirasol, Tomás Cordero

**Affiliations:** Chemical Engineering Department, Andalucía Tech, Universidad de Málaga, 29010 Málaga, Spain; javiertorres@uma.es (J.T.-L.); ramiro@uma.es (R.R.-R.); mirasol@uma.es (J.R.-M.); cordero@uma.es (T.C.)

**Keywords:** methanol dehydration, dimethyl ether, biomass-derived carbon, zirconium phosphate, kinetic modelling, deactivation

## Abstract

A Zr-loaded P-containing biomass-derived activated carbon (ACPZr) has been tested for methanol dehydration between 450 and 550 °C. At earlier stages, methanol conversion was complete, and the reaction product was mainly dimethyl ether (DME), although coke, methane, hydrogen and CO were also observed to a lesser extent. The catalyst was slowly deactivated with time-on-stream (TOS), but maintained a high selectivity to DME (>80%), with a higher yield to this product than 20% for more than 24 h at 500 °C. A kinetic model was developed for methanol dehydration reaction, which included the effect of the inhibition of water and the deactivation of the catalyst by coke. The study of stoichiometric rates pointed out that coke could be produced through a formaldehyde intermediate, which might, alternatively, decompose into CO and H_2_. On the other hand, the presence of 10% water in the feed did not affect the rate of coke formation, but produced a reduction of 50% in the DME yield, suggesting a reversible competitive adsorption of water. A Langmuir–Hinshelwood reaction mechanism was used to develop a kinetic model that considered the deactivation of the catalyst. Activation energy values of 65 and 51 kJ/mol were obtained for DME and methane production in the temperature range from 450 °C to 550 °C. On the other hand, coke formation as a function of time on stream (TOS) was also modelled and used as the input for the deactivation function of the model, which allowed for the successful prediction of the DME, CH_4_ and CO yields in the whole evaluated TOS interval.

## 1. Introduction

Global warming, as well as fossil fuel depletion, are pushing the actual model of energy consumption to a more environmentally friendly scenario [1]. In this new renewable and sustainable environment, the use of waste biomass for the production of chemicals, liquid fuels and advanced catalysts could help to achieve a circular economy and expand the life cycle of the products.

Dimethyl ether (DME), as an interesting renewable diesel substitute, has been widely studied in recent years. Its global market has been increasing and is expected to increase at a compound annual growth rate of 7.5% in the period 2021–2026 [2]. 

DME can be used as liquified petroleum gas-blending, aerosol propellant and low-soot emission diesel substitute [3,4,5]. In addition, DME is an interesting hydrogen vector [6].

The production of renewable DME comes from syngas obtained by biomass gasification. This production of DME can be carried out in two different ways. The first one is the direct route, in which a bifunctional catalyst is used to transform syngas into DME. The most common catalyst used in this is commercial Cu-Zn-Al_2_O_3_, physically mixed with γ-alumina or zeolite [7]. On the other hand, the most widespread process used to industrially obtain DME is the indirect method, in which methanol is synthetized from syngas in the first stage, using a metallic catalyst, and then dehydrated to DME on an acid catalyst. In this sense, methanol dehydration was industrially carried out over γ-alumina [8]. However, other catalysts have shown activity in this process, such as modified HZSM-5 zeolite [9], heteropolyacids [10], polymer resins [11], zirconium phosphate [12] and activated carbons [13,14]. 

Within a sustainable economy, the use of biomass residues as precursor to obtain advanced catalytic materials can result in a positive environmental and economic impact. In this sense, activated carbon, when used as a catalyst or a catalyst support, present several advantages compared to conventional inorganic materials. The porosity of activated carbons can be tailored to cover the special requirements of different applications [15]. They also show a high thermal and chemical stability. Moreover, the control of different operating parameters during the activated carbon synthesis allows for the generation of a favorable surface chemistry, improving the anchoring, immobilization and dispersion of a wide variety of active phases [16]. Additionally, when the catalysts are exhausted, they can be gasified, recovering the active phase, and, at the same time, producing syngas as feedstock for renewable methanol, increasing their lifecycle [17,18].

Carbon materials have been extensively studied in our research group, led by Prof Cordero. Specially, chemically activated carbons with phosphoric acid have shown interesting applications as adsorbents [19,20,21], catalyst supports [22,23,24] and catalysts themselves [14,25,26]. During this activation process, phosphoric acid catalyzes the bond cleavage and formation of phosphate and polyphosphate crosslinks due to cyclization and condensation reactions. The P-related bridges produce a dilation of the carbon structure, leading to a well-developed pore structure after their removal in the washing step. Besides, some phosphorus groups remained well-dispersed on the carbon, and were thermally and chemically stable, giving the carbon a higher resistance to oxidation [27]. Those phosphorus groups, in the form of C-O-P (C-O-PO_3_, (C-O)_2_-PO_2_ or (C-O)_3_-PO) or C-P (C_3_-PO, C_2_-PO_2_, C-PO_3_) groups, have shown a stable 20% yield to DME at 300 °C for more than 20 h in the methanol-to-DME reaction under air flow. However, when oxygen was not present in the reaction medium, conversion decayed to a residual value of around 7% after only 20 min [14]. To overcome this problem, zirconium was added to those P-containing activated carbons, obtaining surface zirconium phosphate species [28,29]. The latter catalyst achieved a stable 50% yield to DME for more than 72 h at 350 °C, without the release of any other byproducts [30]. However, when the temperatures were higher, similar to those used in industrial synthesis, a certain loss of activity with time on stream (TOS) can be detected. This slow deactivation can be associated with the deposition of coke on Zr-O-P-type active sites. C-O-P groups were also present in the catalyst, but those groups were deactivated faster than Zr-O-P ones. Moreover, when the spent catalyst was treated with air at 350 °C for 2 h, only a partial regeneration of the catalyst could be observed, as C-O-P groups were recovered; meanwhile, deactivated Zr-O-P-type active sites remained inactive [29].

A detailed study of the reactions involved can be very useful when optimizing the synthesis of the catalysts. The kinetic study of the dehydration of methanol to DME has previously been reported in the literature for different inorganic catalysts [31,32]. In this sense, determination of the kinetic parameters of this reaction ranges from very simple empirical equations, which only fit experimental data, to mathematical equations as a function of temperature [33], to semiempirical equations [34,35], or even more complex models, based on the reaction mechanisms. Several models are derived from Langmuir–Hinshelwood (LH) [34,36] and Eley–Rideal (ER) mechanisms [37,38,39,40], considering the dissociative or molecular adsorption of methanol [41,42], which could be related to the type of catalyst: LH for γ-Al_2_O_3_ [43], LH and ER for zeolite-type materials [44,45] and LH for SAPO catalysts [46]. In addition, some authors accounted for the influence of water in the inlet stream [47]. 

With regard to the Zr-loaded P-containing carbon catalyst, a mechanism for methanol dehydration at low temperatures was proposed, which is also based on an LH mechanism, where two methanol molecules were adsorbed on one active site, with different adsorption enthalpies, and considering the competitive adsorption of water on the active sites [28]. When that Zr-loaded P-containing carbon was submitted to higher temperatures, a different reaction pathway seemed to take place, because some coke formation was observed. This reaction pathway considered the release of methane and the formation of a formaldehyde intermediate, which could yield water and coke, which were irreversibly adsorbed on the active site, or carbon monoxide and hydrogen, regenerating the active site [29]. 

Coke deposition is one of the main causes of catalyst deactivation in the MTD process. The study of deactivation kinetics under harsh operation conditions is of critical relevance in the development of more stable catalysts. Some semi-theoretical were developed to relate coke production with catalyst deactivation. In this sense, some authors proposed a method for deactivation quantification, which consisted of the addition of an activity factor in every reaction rate affected by the deactivation process [48,49,50,51]. Similar approaches has been widely reported in the literature [50,52,53].

In this work, a complete kinetic model for the methanol-to-dimethyl-ether reaction over a Zr-loaded P-containing carbon catalyst was proposed, which also involved the formation of coke. In addition, a deactivation function was determined as a function of coke content and used to predict the DME yield and methanol conversion decays as a function of time on stream.

## 2. Materials and Methods

### 2.1. Catalyst Preparation

Olive stone was used as raw material to prepare the catalyst support. Olive stone, an abundant and low-cost biomass waste from the olive oil industry, was supplied by Sociedad Cooperativa Andaluza Olivarera y Frutera San Isidro, Periana (Málaga), Spain. The olive stone was initially impregnated with phosphoric acid (H_3_PO_4_, 85% *w/w*, Panreac, Castellar del Vallés, Spain) at a mass ratio 2/1 (H_3_PO_4_/olive stone). After that, it was dried overnight, at 60 °C, in an oven. The mixture was introduced in a tubular furnace under a 150 cm^3^/min N_2_ (99.999%, Linde, Barcelona, Spain) flow and the temperature was raised at a heating rate of 10 °C/min to 800 °C, at which the sample remained for 2 h. Then, the sample was washed with distilled water, at 60 °C, until a constant pH was reached in the residual water, and sieved, between 100 and 300 μm. The chemically activated carbon obtained (ACP) was, subsequently, impregnated by the incipient wetness method with zirconium (IV) oxynitrate hydrate (N_2_O_7_Zr·xH_2_O, 99%, Sigma Aldrich, St. Louis, MO, USA). The amount of salt used was calculated to obtain a zirconium mass loading of 5.25%. The impregnated sample was then dried at 120 °C overnight and heated at 250 °C for 2 h, in a muffle furnace. A more detailed description of the catalyst preparation process can be found elsewhere [30].

### 2.2. Characterization

The textural properties of the catalyst were analyzed by N_2_ adsorption–desorption at −196 °C in an ASAP 2020 instrument (Micromeritics, Norcoss, GA, USA). The sample was outgassed for at least 8 h, at 150 °C, before the analysis. From the N_2_ isotherm data, apparent surface area (A_BET_) was obtained by the Brunauer, Emmett and Teller (BET) equation [54]; micropore volume (V_t_) and external surface area (A_t_) were calculated by t-method [55]; and mesopore volume (V_mes_) was obtained as the difference between the volume adsorbed (V_tot_) at a relative pressure close to unity (0.995) and micropore volume [56]. From the CO_2_ isotherm, narrow micropore volume (V_DR_) and narrow surface area (A_DR_) were calculated by applying the Dubinin–Radushkevich equation [57].

The surface chemistry of the catalyst was studied by X-ray photoelectron spectroscopy (XPS). This analysis was carried out in a spectrophotometer PHI 5000 VersaProbe II (Physical Electronics, Chanhassen, MN, USA), with MgK α radiation (1253.6 eV). C_1s_ peak was located at 284.5 eV and used as a reference to place the rest of the peaks.

### 2.3. Catalyst Performance

Methanol dehydration was carried out using pure methanol (CH_3_OH, purity 99.9%, Carlo Erba, Sabadell, Spain) or a mixture of methanol and distilled water. The experiments were carried out in a fixed-bed microreactor (4 mm i.d.) located in a vertical furnace, working under atmospheric pressure. Methanol or methanol and water were fed to the reactor using a syringe pump (Cole-Parmer^®^ 74900-00-05 model, Cole-Parmer Instrument Company, Vernon Hills, IL, USA), using a 70 cm^3^/min nitrogen flow (purity 99.999%, Linde). The reaction temperature ranged from 450 °C to 550 °C, while the reactant partial pressure varied from 0.015 to 0.08 atm and the catalyst mass from 50 to 300 mg, obtaining spacetime values from 50 to 100 g_cat_·s/mmol_reactant_. All the pipelines were heated at 120 °C to avoid methanol or any other product condensation.

Concentrations of gas reactants and products were measured on-line by a Varian CP-4900 gas micro-chromatograph (Agilent Technologies Spain, Madrid, Spain), equipped with capillary columns: 5A molsieve, PPQ and wax columns. This equipment allowed the gas outlet concentration to be sampled every 4 min. Coke content was quantified by direct weighing of the catalyst before and after reaction, and coke selectivity was calculated by assuming that the produced coke consisted of pure carbon.

Conversion, selectivity and yield were defined by the following expressions:

(1) $$X=\frac{F_{0}-F_{\;}}{F_{0}}$$

(2) $$S=\frac{n_{i}\cdot F_{i}}{\sum n_{i}\cdot F_{i}}$$

(3) y=X·S

where X represents the conversion, S the selectivity and y the yield. $F_{0}$ is the reactant molar flow fed to the reactor; F is the reactant molar flow at the outlet stream; F_i_ stands for the molar flow of the product i at the outlet stream and n_i_ represents the number of carbon atoms in the corresponding i molecule. 

## 3. Results and Discussion

### 3.1. Catalyst Properties

Table 1 shows the textural parameters obtained from N_2_ adsorption isotherm at −196 °C and CO_2_ adsorption isotherm at 0 °C.

The catalyst exhibited an A_BET_ value of 1105 m^2^/g, this value is much higher than other inorganic catalysts reported in the literature [8,42,45]. In addition, the contribution of mesopores to the total pore volume is also very significant (almost 50%), which is favorable for catalytic reactions. On the other hand, the micropore volume measured by N_2_, V_t_ was more than twice the micropore volume determined by CO_2_ adsorption, V_DR_, which indicated a high preponderance of wide microporosity [21]. Figure S1 shows the N_2_ adsorption–desorption isotherm at −196 °C of the fresh catalyst, which can be associated with a type IV(a) isotherm with a H4 hysteresis loop, evidencing the presence of in-bottle shape mesopores [56].

Table 1 also shows the atomic surface concentration obtained from XPS analysis. Carbon and oxygen were the main compounds detected on the surface, but phosphorus and zirconium were also observed. As can be seen, the P/Zr ratio was close to 1. This ratio was lower than the theoretical ratio found in zirconium phosphate species, suggesting the possible formation of other Zr surface groups, which were not bounded to phosphorus. In this sense, Figure S2 shows the individual XPS spectra of P_2p_ and Zr_3d_ of the catalyst. The P_2p_ spectrum (Figure S2a) showed a broad band associated with the presence of different phosphorus compounds, such as C_3_PO, C−PO_3_/C_2_PO_2_, C−O−PO_3_ and zirconium phosphate surface groups, as in Zr(HPO_4_)_2_·H_2_O. On the other hand, the Zr_3d_ spectrum (Figure S2b), also presents a broad band, mainly attributed to the presence of zirconium–phosphate groups and, to a lesser extent, Zr−carbon/ZrO_2_ species and Zr (IV) bound to an electroactive species, such as pyrophosphate groups. 

### 3.2. Catalyst Performance

**Figure 1:**
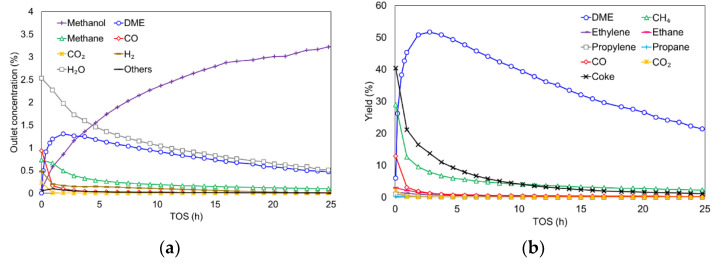
Gas outlet concentration (**a**) and yield to different products (**b**) as a function of TOS in the MTD reaction. Reaction conditions: temperature of 500 °C, methanol partial pressure of 0.04 atm and a space time of 75 g_cat_·s/mmol_CH3OH_.

This Zr-loaded P-containing biomass-derived catalyst was already tested at temperatures below 400 °C for the methanol to DME (MTD) reaction (0.02 atm_CH3OH_ and space time of 75 g_cat_·s/mmol_CH3OH_) [30]. This catalyst showed a stable and selective DME production for more than 24 h. However, its performance at higher temperatures, where the methanol conversion is very high (100%), closer to that of the industrial process, was evaluated in this work. Figure 1a shows the gas outlet concentration and Figure 1b represents the product yields as a function of TOS at 500 °C, 0.04 atm_CH3OH_ and a space–time of 75 g_cat_·s/mmol_CH3OH_.

As can be seen, the initial methanol concentration is near to zero (very high methanol conversion), with value being even higher than that predicted by methanol-DME equilibrium, possibly due to the presence of side reactions. At a higher TOS, the methanol concentration slowly increased. In spite this decrease in conversion, methanol conversion was kept higher than 25% for more than 24 h. This decay in conversion was attributed to the catalyst deactivation caused by the formation of coke. 

The main gas products obtained in this reaction were DME, water, methane, CO and hydrogen, with only traces of CO_2_, ethane, ethylene, propane and propylene. DME yield reached a maximum around 50% at 3 h. After that TOS, DME yield gradually reduced. Nevertheless, that yield remained higher than 20% for more than 24 h, evidencing the high selectivity of this catalyst to DME (around 85%), even under these operating conditions. In this sense, the yield to coke and methane was initially significant, but they did not exceed 10% after 5 h. The CO and hydrogen evolution was similar to that found for methane and coke. However, the concentration of water did not present the same tendency as that of DME at the earlier TOS, suggesting that water is also produced by other side-reactions, associated with the formation of coke and methane.

The same trends can be observed at different reaction temperatures (450 °C and 550 °C), concentrations (1.5% and 8%) and spacetimes (50 g_cat_·s/mmol_CH3OH_ and 100 g_cat_·s/mmol_CH3OH_), as can be seen in Figure S3. 

To explain the main product distribution shown in Figure 1, the following reactions were considered: methanol dehydration to DME (Equation (4)); methane formation, which takes into account the additional formation of water (Equation (5)); CO formation (Equation (6)); and coke production (Equation (7)) [29].

(4) $$2{\;\mathrm{CH}}_{3}{\mathrm{OH}}_{\left(\mathrm{ads}\right)}\to {\mathrm{CH}}_{3}{\mathrm{OCH}}_{3}+H_{2}O$$

(5) $$2{\;\mathrm{CH}}_{3}{\mathrm{OH}}_{\mathrm{ads}}\to {\mathrm{CH}}_{4}+H_{2}O_{\left(\mathrm{ads}\right)}+{\mathrm{CH}}_{2}O_{\left(\mathrm{ads}\right)}$$

(6) $${\mathrm{CH}}_{2}O_{\left(\mathrm{ads}\right)}\to \mathrm{CO}+H_{2}$$

(7) $${\mathrm{CH}}_{2}O_{\left(\mathrm{ads}\right)}\to C_{\mathrm{coke}}+H_{2}O$$

The coke production was considered to take place due to an intermediate similar to formaldehyde, as some authors have already reported [58]. This formaldehyde would instantaneously decompose under the operating conditions used in this study, yielding CO and H_2_ or coke and water. To validate this assumption, an experiment was carried out in which methanol was cofed with formaldehyde and water, and this was compared to an experiment in which the same partial pressure of methanol and water were added without formaldehyde. Figure S4 collects the coke content as a function of TOS at 500 °C and 75 g_cat_·s/mmol_CH3OH_, cofeeding 4% methanol and 2% water without and with 1.5% of formaldehyde. The quantity of coke deposited on the catalyst was 15% higher in the presence of formaldehyde in the inlet stream, suggesting the significant role of formaldehyde in the formation of coke. 

#### 3.2.1. Effect of Inlet Water Vapor

**Figure 2:**
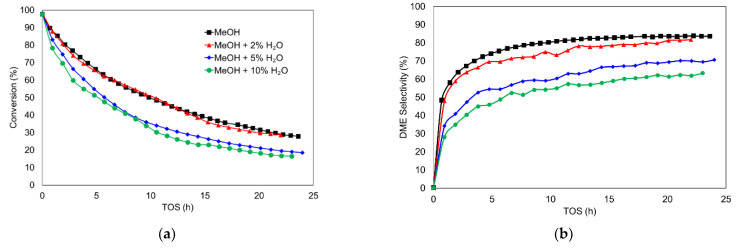
Methanol conversion (**a**) and DME selectivity (**b**) as a function of TOS in the MTD reaction with different cofed water concentrations. Reaction conditions: temperature of 500 °C, methanol partial pressure of 0.04 atm and a space time of 75 g_cat_·s/mmol_CH3OH_.

Water has been reported to compete with methanol for the active sites of the catalyst, causing a decay in DME production [30,59,60,61]. The inhibitory effect of water is relevant in the experimental conditions reported here, as its concentration is similar to the concentration of DME observed in this work. In this sense, Figure 2a collects the conversion obtained cofeeding methanol (4%) with different water concentrations.

When water concentration did not exceed 2%, there were no clear signs of decay in the methanol conversion, probably due to the low level of differences between the water produced from methanol dehydration and water cofed with methanol. However, a decrease in conversion rate of around 30% was observed when 5% of water was cofed with methanol, and an even higher decay was noticed when cofed water reached 10%. These data were in concordance with the competitive adsorption of water.

**Figure 3:**
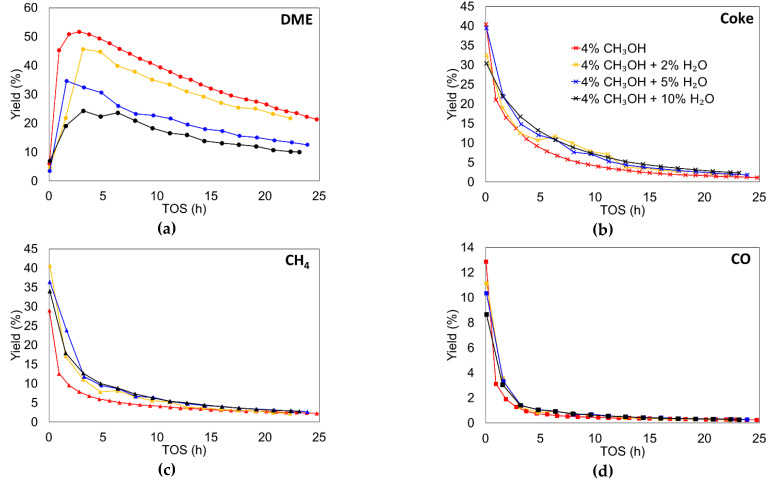
Yield to DME (**a**), Coke (**b**), Methane (**c**) and CO (**d**) as a function of TOS in the MTD reaction with different cofed water concentrations. Reaction conditions: temperature of 500 °C, methanol partial pressure of 0.04 atm and a space time of 75 g_cat_·s/mmol_CH3OH_.

The influence of water was also analyzed in terms of yield and selectivity. Figure 2b shows the selectivity to DME and Figure 3 shows the effect of water concentration on the yields to different products in the MTD reaction.

The inhibitory effect of water can clearly be seen if only selectivity or yield to DME are considered. In that case, the selectivity or yield to DME decreased as water concentration in the reactor inlet increased. In fact, the maximum of DME yield diminished from 52% when only methanol was fed, to 25% when 10% of water was cofed. In this sense, Akarmazyan et al. [8], with a γ-Al_2_O_3_ catalyst, observed a more than 20% decay in methanol conversion after 5 h on stream, when 10% of inlet water was cofed; Xu et al. [61], with a different inorganic solid–acid catalyst, observed a decay in more than 50% when 3% of water was added; Palomo et al. [30], with a Zr-loaded P-containing activated carbon catalyst, reported a reversible activity loss of 10% when 2% of water was cofed with methanol, suggesting, in this case, that the inhibition effect is more related to competitive adsorption than an irreversible deactivation of the active site.

On the other hand, coke yield remained practically the same when water was cofed, suggesting that the mechanism of coke production was not greatly influenced by the presence of water, probably because the main coke precursor was methanol rather than DME (whose concentration decreased in the presence of water). These results are apparently in opposition to other results reported in the literature, where the presence of water reduced the coke production. However, as Gayubo et al. reported [62], this reduction could also be associated, for example, with a lower reactivity due to the dealumination of zeolitic type-catalysts. 

With regard to the formation of methane and CO, their corresponding yields are not affected by the cofeeding of water, independently of the partial pressure of inlet water used, in agreement with the proposed Equations (5)–(7), since coke and CO were probably formed from the formaldehyde intermediate, whose formation implies the simultaneous release of methane, and water is not involved. 

All these results seem to point out that the reduction in methanol conversion when water was cofed, shown in Figure 2, can mainly be attributed to the reduction in the selectivity to DME, since no other relevant changes were observed. In this sense, the equilibrium reaction of methanol dehydration could be shifted to methanol formation from DME in the presence of large quantities of water. 

#### 3.2.2. Stoichiometric Study of MTD Reaction

**Figure 4:**
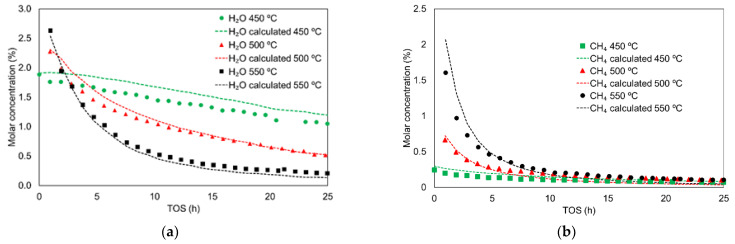
Experimental (symbols) and calculated (dashes lines) molar concentrations of water (**a**) and methane (b) as a function of TOS, at different reaction temperatures. Reaction conditions: methanol partial pressure of 0.04 atm and a spacetime of 75 g_cat_·s/mmol_CH3OH_.

A study on the stoichiometry of the MTD reaction was performed, considering only the main compounds that were obtained, in order to validate the reaction pathway proposed in Equations (4)–(7). The proposed reaction pathway considers that water release was attributed to methanol dehydration (Equation (1)) and coke production (Equation (4)). Olefinsproduction could also produce water, but has been disregarded because they were detected at very low concentrations (traces) compared to the formation of DME and coke. Based on these equations, experimental outlet water concentration must be equal to the water released by DME production (equimolar to DME concentration) plus the water released by coke deposition (equimolar to coke precursor). Figure 4a shows the experimental and calculated water in the MTD at 450 °C, 500 °C and 550 °C. Both profiles are very similar at every TOS, at 500 and 550 °C, and the slight deviation found at 450 °C could be related to the lower coke production observed at this temperature, which can induce more experimental error. In any case, these similarities supported the assumption that water was mainly released by DME and coke production.

On the other hand, methane release seems to be associated with the formation of CH_2_O(ads) intermediate by Equation (5). This CH_2_O(ads), subsequently, evolves to CO (Equation (6)) or coke (Equation (7)). Figure 4b compares the experimental methane concentration with the sum of CO concentration and coke production (equimolar to coke precursor) at different temperatures. A similar tendency can be seen in both concentration profiles, endorsing the reaction pathway proposed in Equations (6) and (7). In addition, it is important to highlight that the formation of methane is very low (concentrations lower than 0.3%) from a TOS of 10 h.

Finally, the similarity between the concentrations of hydrogen and carbon monoxide, observed in Figure 1a, was also in accordance with the reaction proposed in Equation (4), which predicted the equimolecular release of both compounds. The slightly higher release of CO compared to hydrogen evolution can be attributed to the decomposition of oxygen surface groups present on the activated carbon surface, which evolve into CO at the range of temperatures used in this work [63].

### 3.3. Kinetic Study including Deactivation

Considering the aforementioned analysis, a kinetic study was carried out over the ACPZr catalyst for the MTD reaction. With this goal, the reaction pathways previously reported for this catalyst at different experimental conditions were considered. In this sense, Palomo et al. following a Langmuir–Hinshelwood mechanism, proposed a four-step mechanism for DME production, in which two methanol molecules were sequentially adsorbed in the active sites, and then reacted with each other to produce DME and adsorbed water, which was desorbed to regenerate the initial active sites [28]. On the other hand, at higher temperatures (between 450 °C and 550 °C), two adsorbed methanol molecules could also react through a six-member ring, producing methane and an intermediate that mainly evolved into coke and water or CO and hydrogen [29].

For this reason, this model included DME formation, but also coke, methane and CO, as the main carbonaceous byproducts. Nevertheless, olefin and paraffin production were not considered, as they are only found at trace levels in the reactor outlet. 

Initially, the reaction rate at zero-time on stream was predicted by a kinetic study [28,29]. The operating conditions used for the kinetic study were as follows: inlet methanol partial pressure from 0.015 to 0.08 atm and inlet water from 0.02 to 0.1 atm; spacetimes from 50 to 100 g·s/mmol_reactive_; and temperature from 450 °C to 550 °C. The assumptions considered for the development of the kinetic study were:

- A uniform distribution of active sites on the catalyst surface;
- Homogeneous distribution of the catalyst in the catalytic bed;
- Ideal flow, without radial gradients of concentration;
- Isotherm catalytic bed;
- Negligible heat and mass transfer limitations.

For this reason, the plug flow integral reactor can be used to describe the experimental data. The mass balance equation for methanol, DME, methane and CO can be defined in the form of Equation (8)

(8) $$-\frac{{\mathrm{dX}}_{i}}{d\left(\frac{W}{F_{{\mathrm{MeOH}}_{0}}}\right)}=r_{i}$$

where $X_{i}$ is extent of the reaction, which could account for methanol conversion or DME, methane and CO yield; W is the catalyst mass (g); $F_{{\mathrm{MeOH}}_{0}}$ accounts for the inlet methanol molar flow (mol/s) and $r_{i}$ is kinetic rate of consumption or formation for the i species (atm/(g·s)). 

The temperature dependence of kinetic constants, $k_{i}$, is considered to follow Arrhenius law (Equation (9)), while adsorption and equilibrium constants, $K_{i}$, followed Van’t Hoff law (Equation (10))

(9) $$k_{i}=k_{0,i}^{\;}\cdot \exp\left(\frac{-E_{a,i}}{\mathrm{RT}}\right)$$

(10) $$K_{i}=K_{0,i}\cdot \exp\left(\frac{-∆H_{i}}{\mathrm{RT}}\right)$$

where $k_{0,i}^{\;}$ and $K_{0,i}$ are the apparent preexponential factors; R is the universal gas constant (J/mol·K); T is the reaction temperature (K); $E_{a,i}$ is the activation energy of reaction i (J/mol) and $∆H_{i}$ the adsorption enthalpy of equilibrium i. 

A detailed description of the reactions that occur at higher temperatures, (summarized in Equations (4)–(7)), based on the previously described mechanisms, are collected in Equations (11)–(17).

(11) $${\mathrm{CH}}_{3}\mathrm{OH}+*↔{*M}_{\;}$$

(12) $${\mathrm{CH}}_{3}\mathrm{OH}+*M↔M*M\;$$

(13) $$M*M↔*W+{\mathrm{CH}}_{3}{\mathrm{OCH}}_{3}$$

(14) $$*W↔*+H_{2}O$$

(15) $$M*M\to *I+{\mathrm{CH}}_{4}+H_{2}O$$

(16) $$*I↔*+\mathrm{CO}+H_{2}$$

(17) $$*I\to *C+H_{2}O$$

In this scheme, ∗ stands for free active site; ∗M represents one adsorbed methanol molecule on the active site; M∗M means two adsorbed methanol molecules, as described in previous works [28,29]; ∗W corresponds to an adsorbed water molecule; whereas ∗I and ∗C represent a formaldehyde intermediate and coke deposited on the active site, respectively. Equations (11), (12) and (14) represent methanol and water adsorption equilibria, respectively, while Equation (13) accounts for DME formation reaction; Equation (15) represents the formaldehyde intermediate formation with methane and water evolution; Equation (16) represents the decomposition of formaldehyde to CO and hydrogen; and Equation (17) represents the decomposition of formaldehyde intermediate to coke. Moreover, no adsorbed DME step has been taken into account as DME desorption was considered occur very quickly.

Assuming a fast equilibrium for the adsorption of methanol and water, the concentration of adsorbed species can be estimated as follows:

(18) $$C_{*M}=K_{M,1}\cdot P_{M}\cdot C_{*}$$

(19) $$C_{M*M}=K_{M,2}\cdot P_{M}\cdot C_{*M}=K_{M,2}\cdot K_{M,1}\cdot P_{M}^{2}\cdot C_{*}$$

(20) $$C_{*W}=K_{W}\cdot P_{W}\cdot C_{*}$$

where $C_{*}$ is the concentration of free active sites that are available for the adsorption of methanol or water molecules (mol/g); $C_{*M}$ and $C_{M*M}$ are the concentrations of active sites with one or two adsorbed methanol molecules, respectively (mol/g); $C_{*w}$ is the concentration of active sites with adsorbed water; $K_{M,1}$ and $K_{M,2}$ stands for the adsorption equilibrium constants of a single methanol and a second methanol molecule on the active site (atm^−1^); $K_{W}$ represents water equilibrium adsorption constant (atm^−1^); and $P_{M}$ and $P_{W}$ represent the partial pressures of methanol and water (atm). The site balance is described in Equation (21).

(21) $$C_{*t}=C_{*}+C_{*M}+C_{M*M}+C_{*W}+C_{*I}$$

In this, $C_{*t}$ represents the total concentration of active sites (mol/g). This equation can be simplified by assuming that the surface concentration of the intermediate, $C_{*I}$, is negligible compared to the other products, as this specie must be similar to an adsorbed formaldehyde, and this molecule is a highly reactive specie that quickly decomposes into products.

The rate equations for each surface reaction step are shown in Equations (22)–(25)

(22) $$r_{13}={k{}^{\prime }}_{\mathrm{sr}}\cdot C_{M*M}-\frac{{k{}^{\prime }}_{\mathrm{sr}}}{K_{\mathrm{SR}}}\cdot P_{\mathrm{DME}}\cdot C_{*W}$$

(23) $$r_{15}={k{}^{\prime }}_{\mathrm{sr}2}\cdot C_{M*M}$$

(24) $$r_{16}={k{}^{\prime }}_{\mathrm{sr}3}\cdot C_{*I}-\frac{{k{}^{\prime }}_{\mathrm{sr}3}}{K_{\mathrm{SR}3}}\cdot P_{\mathrm{CO}}\cdot P_{H_{2}}\cdot C_{*}={k{}^{\prime }}_{\mathrm{sr}3}\cdot C_{*I}-\frac{{k{}^{\prime }}_{\mathrm{sr}3}}{K_{\mathrm{SR}3}}\cdot P_{\mathrm{CO}}^{2}\cdot C_{*}$$

(25) $$r_{17}={k{}^{\prime }}_{\mathrm{sr}4}^{\;}\cdot C_{*I}$$

where ${k{}^{\prime }}_{\mathrm{sr}}$, ${k{}^{\prime }}_{\mathrm{sr}2}$, ${k{}^{\prime }}_{\mathrm{sr}3}$ and ${k{}^{\prime }}_{\mathrm{sr}4}$ are the kinetic constants of the surface reactions (s^−1^); $K_{\mathrm{SR}}$ (atm^1^), $K_{\mathrm{SR}3}$ (atm^2^) are the equilibrium constant of surface reactions; $P_{M}$, $P_{\mathrm{DME}}$, $P_{W}$, $P_{\mathrm{CO}}$, $P_{H_{2}}$ are the partial pressures of methanol, DME, water, CO and H_2_ (atm), respectively; and $C_{*I}$ is the surface concentration for the intermediate species, presumably adsorbed formaldehyde, that formed on the catalytic site (mol/g). $r_{16}$ was simplified, assuming that the partial pressure of hydrogen must be equal to CO’s partial pressure, since it is the only reaction in which CO and H_2_ are formed, and they are obtained in an equimolecular ratio. This assumption is verified in Figure 1a. Thus, the surface concentration of the intermediate was calculated assuming that $r_{15}$ is equal to $r_{16}+r_{17}$:

(26) $$C_{*I}=\frac{{k{}^{\prime }}_{\mathrm{sr}2}\cdot K_{M,1}\cdot K_{M,2}\cdot P_{M}^{2}+\frac{{k{}^{\prime }}_{\mathrm{sr}3}}{K_{\mathrm{SR}3}}\cdot P_{\mathrm{CO}}^{2}}{{k{}^{\prime }}_{\mathrm{sr}3}+{k{}^{\prime }}_{\mathrm{sr}4}}\cdot C_{*}$$

Combining the site balance equation, Equation (21), with the surface concentration equations, Equations (18)–(20) and (26), the site balance can be rearranged to provide an expression of the free fraction of active sites, $\theta_{*},$ as Equation (27) shows:

(27) $$\frac{C_{*}}{C_{*t}}=\theta_{*}=\frac{1}{1+K_{M,1}\cdot P_{M}+K_{M,1}\cdot K_{M,2}\cdot P_{M}^{2}+K_{W}\cdot P_{W}}$$

If surface reactions are considered as rate-determining steps for every compound, rate expressions for DME (Equation (28)), ${\mathrm{CH}}_{4}$ (Equation (29)) and CO (Equation (30)) can be obtained by substituting the fractional coverage values, as defined in Equations (18)–(20) and (27).

(28) $$r_{\mathrm{DME}}=r_{13}=\frac{k_{\mathrm{sr}}\cdot \left(K_{M,1}K_{M,2}P_{M}^{2}-\frac{P_{\mathrm{DME}}\cdot K_{W}\cdot P_{W}}{K_{\mathrm{sr}}}\right)}{1+K_{M,1}P_{M}+K_{M,1}K_{M,2}P_{M}^{2}+K_{W}\cdot P_{W}}$$

(29) $$r_{{\mathrm{CH}}_{4}}=r_{16}=\frac{k_{\mathrm{sr}2}\cdot K_{M,1}\cdot K_{M,2}\cdot P_{M}^{2}}{1+K_{M,1}P_{M}+K_{M,1}K_{M,2}P_{M}^{2}+K_{W}\cdot P_{W}}$$

(30) $$r_{\mathrm{CO}}=r_{17}=\frac{\frac{k_{\mathrm{sr}2}\cdot k_{\mathrm{sr}3}\cdot K_{M,1}\cdot K_{M,2}\cdot P_{M}^{2}}{{\mathrm{ksr}}_{3}+k_{\mathrm{sr}4}}+\left(\frac{k_{\mathrm{sr}3}}{k_{\mathrm{sr}3}+k_{\mathrm{sr}4}}-k_{\mathrm{sr}3}^{-1}\right)\cdot P_{\mathrm{CO}}^{2}}{1+K_{M,1}P_{M}+K_{M,1}K_{M,2}P_{M}^{2}+K_{W}\cdot P_{W}}$$

In these, all the kinetic constants (from $k_{\mathrm{sr}}$ to $k_{\mathrm{sr}4}$, mol·g^−1^·s^−1^) are redefined as the product of $k_{i}{}^{\prime }\cdot C_{*t}$. Finally, the methanol decomposition rate can be described by attending to the formation rates of the products and considering the stoichiometry of the MTD reaction as follows:

(31) $$r_{{\mathrm{CH}}_{3}\mathrm{OH}}=-2\cdot r_{\mathrm{DME}}-r_{{\mathrm{CH}}_{4}}$$

To obtain the kinetic parameters, a MATLAB^®^ program based on the Nelder–Mead simplex algorithm, was used. The program minimized the objective function (Equation (32)), defined as the square difference between experimental and calculated data for every experiment

(32) $$\mathrm{OF}=\overset{\;}{\underset{\;}{\sum }}{\left(x_{i,\exp}^{\;}-x_{i,\mathrm{cal}}^{\;}\right)}^{2}$$

where $x_{i,\exp}^{\;}$ is the experimental conversion/yield of the i species, and $x_{i,\mathrm{cal}}^{\;}$ is the conversion yield of the i species, estimated from the solution to their respective mass balance equation.

**Figure 5:**
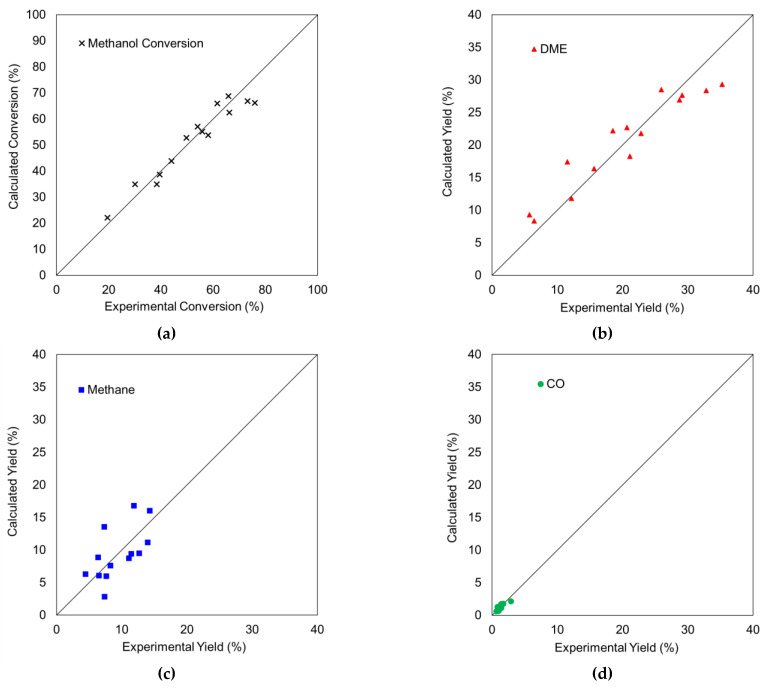
Calculated versus experimental conversion of methanol (**a**) and yield to DME (**b**), Methane (**c**) and CO (**d**) at zero-time on stream for the different operating conditions (temperatures of 450 °C, 500 °C and 550 °C; methanol partial pressure of 1.5%, 4% and 8%; space time of 50, 75 and 10 g_cat_·s/mmol_CH__3__OH_).

Figure 5 compares the calculated data versus the experimental results at zero time on stream, under different operation conditions. As can be seen, the model successfully reproduced the experimental data. Only low deviations were found for methane yield obtained at high methanol partial pressures, where the model overpredicted the methane conversion. Additionally, only low deviations were found for the DME yields and methanol conversions obtained at the lowest temperature (450 °C) and the shortest spacetime (50 g·s/mol_CH3OH_). For the latter case, it looks like the inhibition effect of water in DME formation via the promotion of DME hydration is stronger in the catalyst than the one predicted by the model.

Table 2 collects the kinetic data at zero-time on stream for the reactions proposed in the mechanism. As can be seen, kinetic parameters related to DME formation ($k_{\mathrm{sr}}$, $K_{M,1}$ and $K_{M,2}$) and water desorption ($1/K_{W}$) are very similar to the kinetic parameters proposed by Palomo et al. [28] at lower temperatures, which corroborate that the same mechanism of DME production takes place at the higher temperatures used in this study. Only some discrepancies can be seen for $K_{\mathrm{SR}}$, as the high preexponential factor and low adsorption enthalpy reported in their work produced a negligible value for the DME hydration reaction. The authors stated that the reverse DME formation reaction can be considered negligible for the operation conditions used in their study [28]. Nevertheless, at the conditions tested here, this reaction plays an important role, as discussed in Section 3.2.1. It is also noteworthy that, under these experimental conditions, methanol adsorption on the active site is favored over water adsorption, as suggested by the higher value of $K_{M,1}$ compared to that of $K_{W}$, explaining why coke formation was barely affected by water adsorption (see Table 2). Thus, the inhibitory effect of water on DME formation seems to be established via the DME hydrolysis reaction. The evaluation of DME formation rate and the reverse reaction rate (at 500 °C, a methanol partial pressure of 0.04 atm and methanol conversion of 50%) shows reaction rate values of 1.14 × 10^−5^ and 4.02 × 10^−6^ atm/g·s, respectively. These results suggest that, even in the absence of water in the inlet stream, nearly 40% of the DME would react to produce methanol under these conditions.

On the other hand, activation energy for $k_{\mathrm{sr}2}$ (methane formation) has a lower value than DME formation, which implies that the deactivation rate (triggered by reaction $r_{18}$, which runs in series with methane formation) is less sensitive to temperature changes. This result indicates that DME formation prevails over deactivation by coke at the highest reaction temperature. Finally, the $k{}^{\prime }$ value (which corresponded to the ratio $k_{\mathrm{sr}4}/k_{\mathrm{sr}3}$, and was related to the selectivity towards coke formation in the decomposition of the intermediate) indicates that the activation energy for coke production is higher. Again, this result is in accordance with the experimental data, where the coke formation rate becomes faster than CO formation as the reaction temperature increases. 

Several kinetic studies have been performed for the MTD reaction. Specifically, Hosseininejad et al. [38], with an Amberlist 35 as a catalyst, reported an activation energy of 98 kJ/mol at temperatures between 110 and 135 °C. Lower values of E_a_, around 75 kJ/mol, were proposed by Bercic et al. [34] using γ-Al_2_O_3_, at temperatures between 320 and 360 °C. This value was similar to the E_a_ reported by Mollavali et al. [43], for another γ-Al_2_O_3_, ranged between 57 and 62 kJ/mol, but this was far from the value provided by Sierra et al. [47], 264 kJ/mol, for the same reaction. Migliori et al. [31] with an H-FMI catalyst, proposed different activation energy values, between 50 and 68 kJ/mol, at temperatures between 170 °C and 250 °C, and they related these values to the Si/Al ratio of the zeolite. Pop et al. [46], with H-SAPO-34, obtained an activation energy value of 80 kJ/mol at temperatures between 80 and 250 °C. On ZSM-5, Ortega et al. [45] obtained a E_a_ between 80 and 130 kJ/mol. Ha et al. [44], with a modified ZSM-5, obtained an activation energy of around 55 kJ/mol. With activated carbons, Moreno-Castilla et al. [13] obtained an increasing E_a_ from 85–115 kJ/mol in the temperature range 140–180 °C. Finally, Palomo obtained an E_a_ of 70 kJ/mol for the same catalyst, operating at lower temperatures. Many of these values are similar to the E_a_ obtained in the present kinetic study for the MTD reaction.

All the above rates only account for the zero-time on stream, so they are considered initial reaction rates. As the time on stream increased, some loss of activity was observed, associated with the coke deposition on the active sites. Therefore, to describe the evolution of reactant and product yields with TOS, a deactivation function became necessary. This deactivation function was directly related to the amount of coke that was deposited, which was described by several empirical equations, proposed by Froment et al. [64]. Once the equation describing the coke evolution with TOS is obtained, it will be used as the model input for the catalyst deactivation function, which relates coke formation with catalyst deactivation. The empirical equations that were tested for the coke evolution with TOS are collected in Equations (33)–(37)

(33) $$C_{\mathrm{coke}}=\frac{1}{\alpha }\ln\left(1+\alpha \cdot r_{\mathrm{coke}}^{0}\cdot \mathrm{TOS}\right)$$

(34) $$C_{\mathrm{coke}}=\frac{1}{\alpha }\left[1-\exp\left(-\alpha \cdot r_{\mathrm{coke}}^{0}\cdot \mathrm{TOS}\right)\right]$$

(35) $$C_{\mathrm{coke}}=\frac{1}{\alpha }\left[1-\frac{1}{1+\alpha \cdot r_{\mathrm{coke}}^{0}\cdot \mathrm{TOS}}\right]$$

(36) $$C_{\mathrm{coke}}=\frac{1}{\alpha }\left[\sqrt{2\alpha \cdot r_{\mathrm{coke}}^{0}\cdot \mathrm{TOS}+1}-1\right]$$

(37) $$C_{\mathrm{coke}}=\frac{1}{\alpha }\left[\sqrt[{3}]{3\alpha \cdot r_{\mathrm{coke}}^{0}\cdot \mathrm{TOS}+1}-1\right]$$

where $C_{\mathrm{coke}}$ stands for the percentage of coke deposited on the catalyst; $r_{\mathrm{coke}}^{0}$ is the initial coke production rate and α is a deactivation constant. To predict the data at different operating conditions, initial coke production $r_{\mathrm{coke}}^{0}$ (without deactivation), was predicted using a pseudo nth-order equation (Equation (38))

(38) $$r_{\mathrm{coke}}^{0}=k^{\prime }\cdot \exp\left(-\frac{E_{a}}{\mathrm{RT}}\right)\cdot P_{{\mathrm{CH}}_{3}\mathrm{OH}}^{n}$$

where $k^{\prime }$ is the preexponential factor; $E_{a}$ is the activation energy of coke production; R is the universal gas constant; T is the temperature (K); $P_{{\mathrm{CH}}_{3}\mathrm{OH}}^{\;}$ is the partial pressure of methanol and n is the reaction order. The coke formed at different times on streams, inlet methanol pressures and reaction temperatures, were experimentally determined by weighing the catalytic bed, and the least-square differences between these quantities and the coke predicted by the different models was minimized using the Nelder–Mead simplex algorithm in Matlab^®^:

(39) $$\mathrm{OF}=\overset{\;}{\underset{\;}{\sum }}{\left(C_{\mathrm{coke},\exp}^{\;}-C_{\mathrm{coke},\mathrm{cal}}^{\;}\right)}^{2}$$

where $C_{\mathrm{coke},\exp}^{\;}$ is the experimental amount of coke that was deposited, and $C_{\mathrm{coke},\mathrm{cal}}^{\;}$ is the amount of coke estimated from the Equations (33)–(37).

Table 3 collects the least-square difference (i.e., value of OF at the end of the minimization procedure) and the best-fitting parameters for the different coke-production equations (Equations (33)–(37)), with the deactivation constant (α) being the one with a higher impact in the prediction of coke content. The minimal value used as an optimization parameter, Equation (39), was achieved with Equation (35), so this equation was selected as the model input for the development of the catalytic deactivation function.

**Figure 6:**
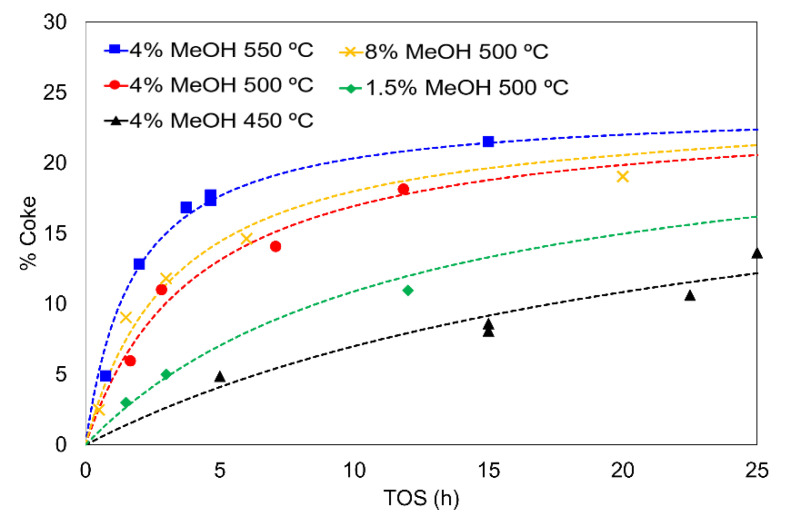
Coke content as a function of TOS for different feeds in the MTD reaction; Reaction conditions: space time of 75 g_cat_·s/mmol_CH3OH_. Experimental data (points) and calculated (lines).

Figure 6 represents the coke content as a function of TOS for the MTD reaction and the calculated coke content by using Equation (35). A good agreement can be seen between experimental and calculated data. Independently of the equation used, all of them predict a reaction order near to one, which is supported by previous works reported in the literature [65]. The activation energy obtained from the fitting (in the range 450–550 °C) has a slightly higher value than values reported for other catalysts under similar conditions [48,50,65], probably because this reaction was performed at higher temperatures (450–550 °C) and the nature of the obtained coke can be different.

Once coke content was successfully modeled as a function of TOS, it was used to obtain a deactivation function, which was added to every rate equation, as shown in Equation (40). The expression of the deactivation function $\Phi_{i}$ is represented in Equation (41)

(40) $$r_{i}=r_{i}^{0}\cdot \Phi_{i}\;{\left(C_{\mathrm{coke}}\right)}_{\;}$$

(41) $$\Phi_{i}=\exp\left(-\alpha_{i}\cdot C_{\mathrm{coke}}\right)$$

where $r_{i}^{0}$ is the rate at zero-time on stream for the i product, i.e., DME, CH_4_ or CO, predicted by the previously described kinetic model, and $\Phi_{i}\left(C_{\mathrm{coke}}\right)$ is the corresponding deactivation function. As can be seen, this deactivation function depends on the coke content, which is a function of TOS and was predicted by Equation (35). Finally, $\alpha_{i}$ is the deactivation constant, which represents the sensitivity of the reaction rate towards deactivation by coke deposition.

The reaction rates for DME, CO and CH_4_ as a function of TOS have been experimentally determined under different operating conditions (temperatures, spacetime, methanol and water inlet pressures) and integrated to obtain the product yields. The least-square differences between the experimental reaction rates and the ones predicted by the coke formation model, considering deactivation (Equations (40) and (41)), were minimized using the aforementioned algorithm previously. 

**Figure 7:**
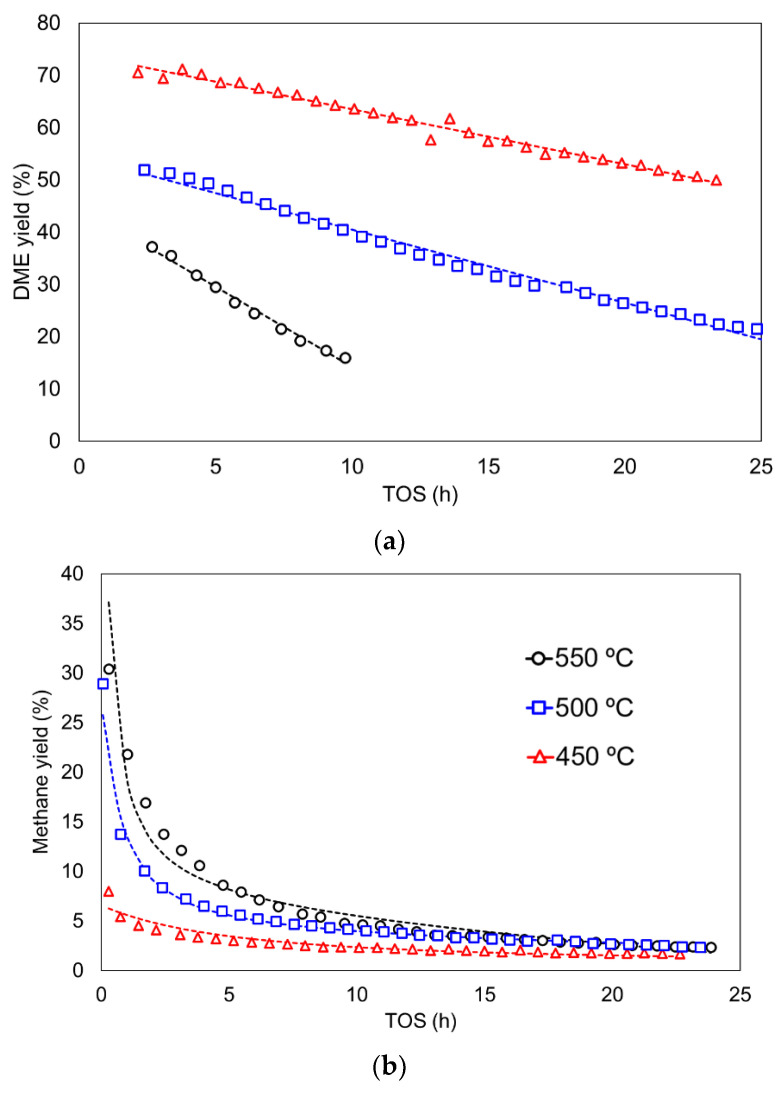

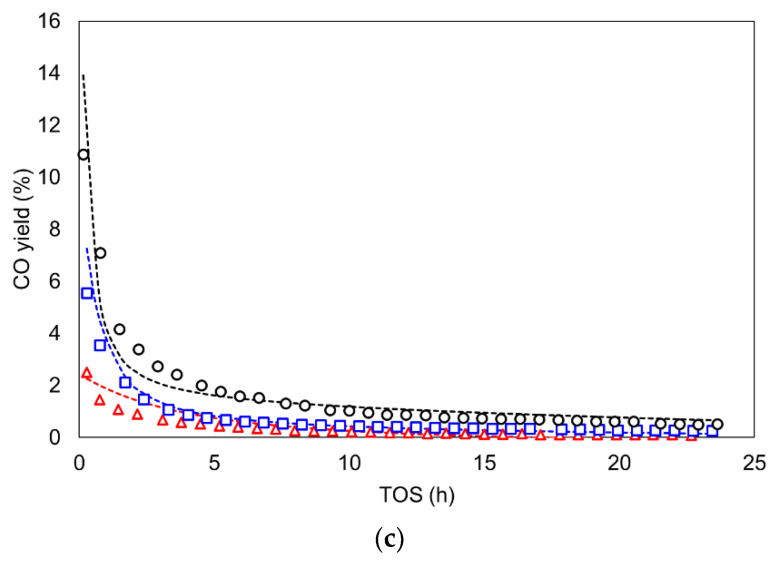
(**a**) DME, (**b**) methane and (**c**) CO yield as a function of TOS at different temperatures. Reaction conditions: methanol partial pressure of 0.04 atm and a space time of 75 g_cat_·s/mmol_CH3OH_.

Figure 7 shows DME, methane and CO experimental yields (points) as a TOS function for different reaction conditions and the calculated ones (lines) that were obtained from the model using the best-fitting parameters. It should be noted that the outlet concentrations showed an induction period, before reaching a semi-steady state, from which point the product yield started to decay with TOS. The model provides a good description of the decrease in the kinetic rate with TOS, showing a strong reduction in yield for CO and methane, and a soft deactivation for DME yields. It is interesting to highlight that the deactivation constant for methane and CO presented rather similar values, 3.18 × 10^−1^; meanwhile, a value of 3.51 × 10^−10^ was obtained for DME. This result clearly points out that methane and CO are produced through the same reaction pathway, which is severely affected by coke content because coke itself is the final product. In contrast, the deactivation of DME production needs a higher amount of coke to achieve the same deactivation degree, and the deactivation is probably caused by indirect mechanisms, such as pore blockage by coke deposition. In this sense, the rate decay for CO and CH_4_ is larger with TOS as temperature increases, whereas DME shows a low sensitivity to the formation of coke. The latter result seems to confirm that the DME reaction pathway is unconnected to coke formation in this catalyst. This clearly differentiated behaviour might be explained if mainly methanol, and not DME, was the main route for coke formation. However, more studies are needed to clarify this point.

## 4. Conclusions

An activated carbon, prepared by the chemical activation of olive stone waste with phosphoric acid, was used as a support of a Zr catalyst. This Zr-loaded P-containing biomass-derived activated carbon catalyst (ACPZr) was tested for methanol dehydration in a wide temperature range, including those temperatures that produce a very high methanol conversion, closer to those used in the industrial process. The catalyst has shown an excellent performance, with high stability and selectivity to DME at temperatures lower than 450 °C. The conversion of methanol was very high (100%) at higher temperatures and reaction products were mainly DME and, to a lesser extent, coke, methane, hydrogen and CO, with DME yields higher than 20%, for more than 24 h at 500 °C. 

A kinetic model that considers the production of the main carbonaceous products (DME, methane, CO and coke) has been proposed, including the inhibitory effect of water. The stoichiometric rates showed that coke could be produced through a formaldehyde intermediate, which can also decompose into CO. The addition of inlet water negatively affects the DME production, with a reduction of around 50% for DME yield when 10% of water was cofed. However, the presence of water did not affect the coke production at temperatures between 450 °C and 550 °C.

The model proposed in this work follows a Langmuir–Hinshelwood mechanism, in which two methanol molecules are adsorbed with different adsorption enthalpy values, with the surface reaction as the rate-determining step. The model successfully predicts methanol conversion as well as DME, methane and CO yields. The activation energy for DME production was around 65 kJ/mol and the activation energy for methane was around 51 kJ/mol, in the range 450–550 °C. The results of the kinetic model led to the conclusion that water inhibition is mainly related to the formation of methanol through the reverse reaction.

On the other hand, coke formation was also modelled as a function of TOS using an empirical equation, which described the coke formation rate very well using an nth-order rate equation, showing an activation energy of 130 kJ/mol and a reaction order close to one. The resulting rate equation of coke formation with TOS was successfully used as the input for the model’s deactivation function, allowing the DME, CH_4_ and CO yields to be successfully predicted in the whole of the evaluated TOS range. The similar deactivation rate values obtained for CH_4_ and CO confirms that both products are related to coke formation. The lower deactivation rate observed for DME might be related to its higher activation energy, which results in a faster increase in the DME rate with temperature.

## Figures and Tables

**Table 1:** Textural parameter values derived from N_2_ adsorption isotherm at −196 °C and CO_2_ adsorption isotherm at 0 °C and atomic surface concentration determined by XPS analysis of the catalyst.

N_2_ Isotherm								CO_2_ Isotherm		
A_t_ (m^2^/g)	A_BET_ (m^2^/g)		V_t_ (cm^3^/g)		V_mes_ (cm^3^/g)	V_tot_ (cm^3^/g)		A_DR_ (m^2^/g)		V_DR_ (cm^3^/g)
279	1105		0.43		0.38	0.80		509		0.20
**Atomic surface concentration (%)**										
C_1s_		O_1s_		P_2p_			Zr_3d_		P/Zr	
65.1		27.0		3.9			3.5		1.11	

**Table 2:** Kinetic parameters of best fit for Equations (28)–(30) and constant value at 500 °C.

Kinetic Parameter	$k_{0}\;or\;K_{0}$	Units	$E_{a}\;or\;\Delta H\;(\mathrm{kJ}/\mathrm{mol})$	Constant Value at 500 °C
$k_{\mathrm{sr}}$	1.3	mol·g_cat_^−1^·s^−1^	65	5.3 × 10^−5^
$K_{\mathrm{SR}}$	5.6 × 10^−11^	atm	−123	0.01
$k_{\mathrm{sr}2}$	2.7 × 10^−2^	mol·g_cat_^−1^·s^−1^	51	9.2 × 10^−6^
$k_{\mathrm{sr}3}^{-1}$	6.6	mol·g_cat_^−1^·s^−1^	15	0.7
${k{}^{\prime }}_{\;}$	69.5	-	19	3.7
$K_{M,1}$	3.0	atm^−1^	−15	31.1
$K_{M,2}$	3.6	atm^−1^	−10	17.1
$K_{W}$	0.01	atm^−1^	−41	8.5

**Table 3:** Parameters for coke deposition using different deactivation equations obtained for temperatures between 450 °C and 550 °C.

Deactivation Equation	α	$k_{c}^{{}^{\prime }}$	$E_{a}\;(\mathrm{kJ}/\mathrm{mol})$	n	OF
(33)	0.184	1.24 × 10^10^	135	0.88	0.019
(34)	0.043	5.14 × 10^8^	122	0.96	0.021
(35)	0.038	2.61 × 10^9^	130	0.79	0.015
(36)	5.98 × 10^14^	3.20 × 10^21^	87	0.88	0.053
(37)	4.16 × 10^7^	1.91 × 10^25^	120	0.98	0.030

## Supplementary Materials

The following are available online at https://www.mdpi.com/article/10.3390/ma15020596/s1, Figure S1. N_2_ adsorption-desorption isotherm at −196 °C of fresh catalyst; Figure S2. XPS spectra of (a) P_2p_ and (b) Zr_3d_ of fresh catalyst; Figure S3. Yield to different products as a function of TOS at different operating conditions in the MTD reaction. (a) 450 °C, 0.04 atm_CH3OH_ and 75 g_cat_/mmol_CH3OH_ (b) 550 °C, 0.04 atm_CH3OH_ and 75 g_cat_/mmol_CH3OH_ (c) 500 °C, 0.015 atm_CH3OH_ and 75 g_cat_/mmol_CH3OH_ (d) 500 °C, 0.08 atm_CH3OH_ and 75 g_cat_/mmol_CH3OH_ (e) 500 °C, 0.04 atm_CH3OH_ and 50 g_cat_/mmol_CH3OH_ (f) 500 °C, 0.04 atm_CH3OH_ and 100 g_cat_/mmol_CH3OH_; Figure S4. Coke content as a function of TOS for methanol+water fed and methanol+water+formaldehyde; temperature 500 °C and spacetime of 75 gcat·s/mmol_CH3OH_. Experimental data (points) and calculated (lines).
