# Supplementary material for: A Kinetic Model Considering Catalyst Deactivation for Methanol-to-Dimethyl Ether on a Biomass-Derived Zr/P-Carbon Catalyst
Source: Materials (Basel). 2022 Jan 13;15(2):596. doi: 10.3390/ma15020596 (PMC8781041; doi:10.3390/ma15020596)
Supplement: Supplementary file 1 [file materials-15-00596-s001.zip › materials-1517280-supplementary.pdf]

materials

Article

A Kinetic Model Considering Catalyst Deactivation for Methanol-to-Dimethyl Ether on a Biomass-Derived Zr/P-Carbon Catalyst

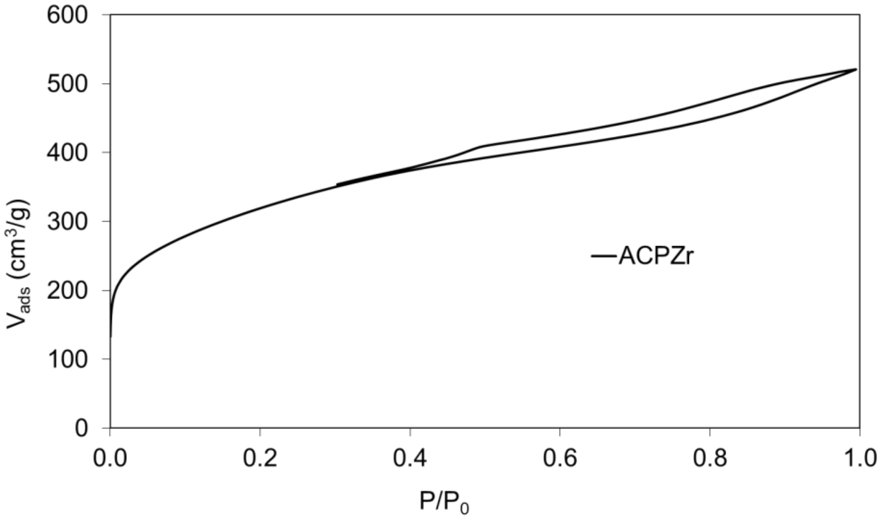

Figure S1. N2 adsorption-desorption isotherm at -196 °C of fresh catalyst.

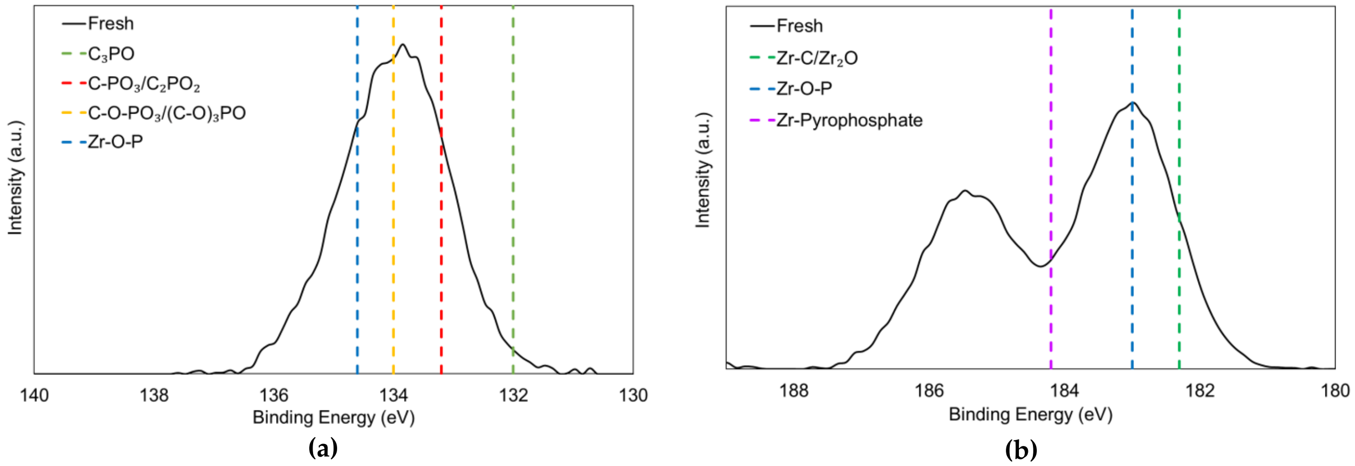

Figure S2. XPS spectra of (**a**) P2p and (**b**) Zr3d of fresh catalyst.

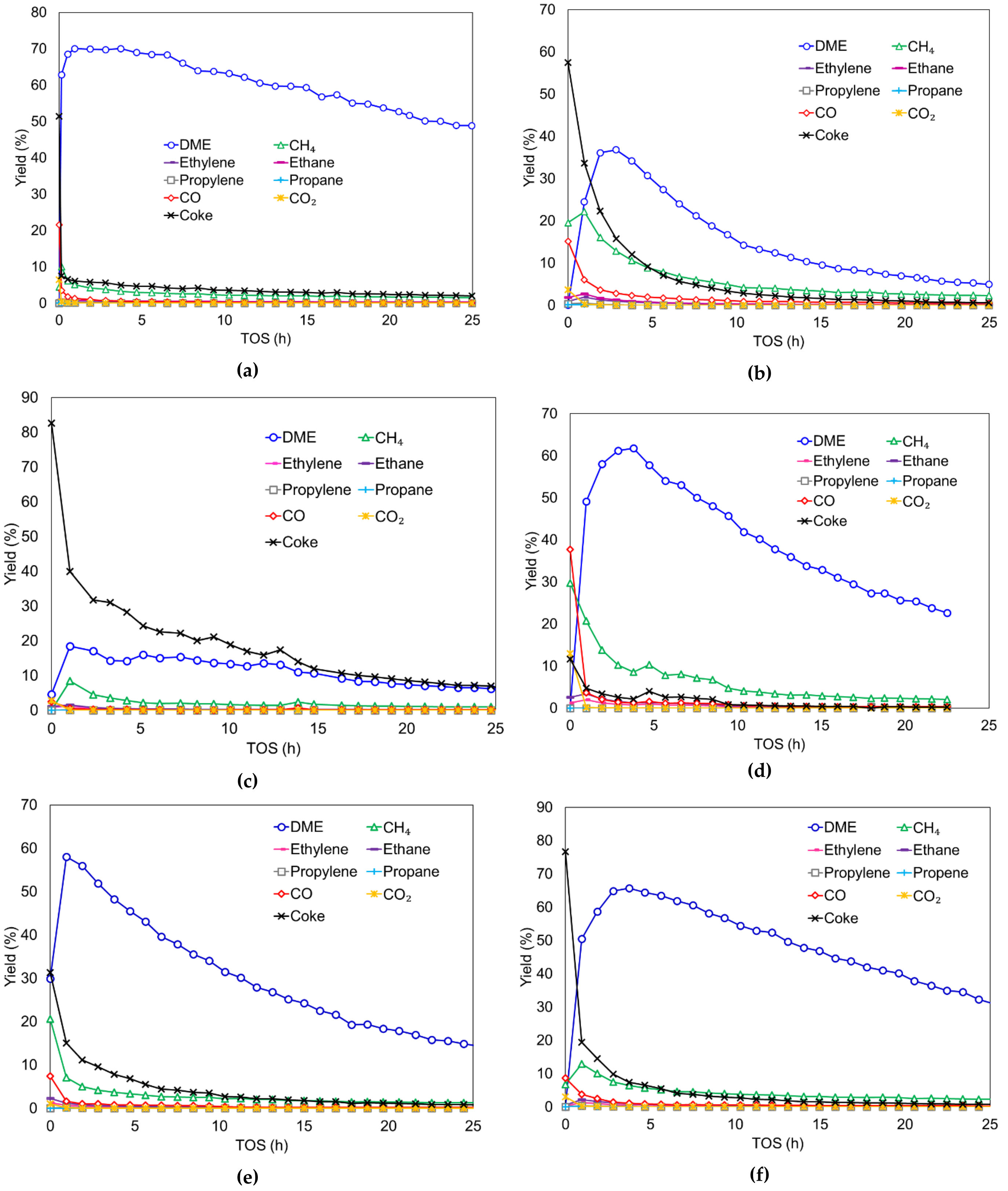

Figure S3. Yield to different products as a function of TOS at different operating conditions in the MTD reaction. (**a**) 450 °C, 0.04 atm$_{CH_3OH}$ and 75 g$_{cat}$/mmol$_{CH_3OH}$ (**b**) 550 °C, 0.04 atm$_{CH_3OH}$ and 75 g$_{cat}$/mmol$_{CH_3OH}$ (**c**) 500 °C, 0.015 atm$_{CH_3OH}$ and 75 g$_{cat}$/mmol$_{CH_3OH}$ (**d**) 500 °C, 0.08 atm$_{CH_3OH}$ and 75 g$_{cat}$/mmol$_{CH_3OH}$ (**e**) 500 °C, 0.04 atm$_{CH_3OH}$ and 50 g$_{cat}$/mmol$_{CH_3OH}$ (**f**) 500 °C, 0.04 atm$_{CH_3OH}$ and 100 g$_{cat}$/mmol$_{CH_3OH}$.

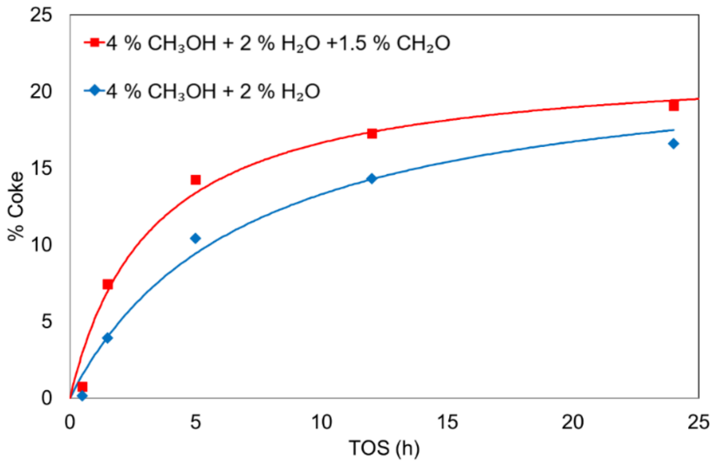

Figure S4. Coke content as a function of TOS for methanol + water fed and methanol + water + formaldehyde; temperature 500 °C and space time of 75 gcat·s/mmolCH3OH. Experimental data (**points**) and calculated (**lines**).
